# *Timareteposteria*, a new cirratulid species from Korea (Annelida, Polychaeta, Cirratulidae)

**DOI:** 10.3897/zookeys.806.27436

**Published:** 2018-12-13

**Authors:** Hyun Ki Choi, Hana Kim, Seong Myeong Yoon

**Affiliations:** 1 National Marine Biodiversity Institute of Korea, Seocheon, Chungcheongnam-do 33662, Korea National Marine Biodiversity Institute of Korea Seocheon Korea, South; 2 Department of Biology, College of Natural Sciences, Chosun University, Gwangju 61452, Korea Chosun University Gwangju Korea, South

**Keywords:** Korean waters, polychaete, taxonomy, *
Timarete
*, COI, 16S

## Abstract

A new cirratulid species, *Timareteposteria***sp. n.**, is described from the intertidal habitats of the eastern coast of South Korea. The new species is closely related to *Timareteluxuriosa* (Moore, 1904) from southern California based on morphological similarity of the branchial and tentacular finalents and the noto- and neuropodial spines. However, *T.posteria***sp. n.** differs from the latter based on the following characteristics: 1) evenly divided peristomium into three annulations; 2) 2–4 neuropodial spines originating in the posterior chaetigers alternated by a few capillaries; and 3) complete shift in branchial finalents located about one-third between the notopodium and the dorsal midline. The new species has a methyl green staining pattern (MGSP) distinct from other *Timarete* species. Detailed description and illustrations of the new species are provided with molecular information based on the partial sequences of the mitochondrial cytochrome c oxidase subunit I (COI) and 16S ribosomal RNA (16S). This study also includes a key and discussion of known *Timarete* species from East Asia.

## Introduction

The genus *Timarete* Kinberg, 1866 is a typical multi-tentaculate genus assigned to the cirratulid polychaetes (Blake 1996). *Timarete* species are clearly distinguished from other multi-tentaculate species by having chaetae including capillaries and acicular spines, tentacular finalents forming a transverse series arising from two or more chaetigers, and branchiae originating in the segment anterior to tentacular finalents and shifted toward the mid-dorsum of the body in middle and posterior chaetigers (Fauchald 1977, Blake 1996).

Recently, several species belonging to *Timarete* have been taxonomically re-evaluated (Blake 1996, Çinar 2007, Magalhães and Bailey-Brock 2010, Magalhães et al. 2014). Blake (1996) redescribed *Timareteperbranchiata* (Chamberlin, 1918) from California and confirmed the taxonomic state of *T.convergens* (Chamberlin, 1918) as a junior synonym of it. Blake (1996) also determined that *Cirriformialuxuriosa* (Moore, 1904), which had been misconstrued and redescribed it as a species of *Timarete*. Çinar (2007) redescribed *Timaretepunctata* (Grube, 1859) and designated a lectotype and paralectotypes based on syntypes from the U. S. Virgin Islands (Caribbean Sea) deposited in the Zoological Museum of Copenhagen, and he also examined materials from the Mediterranean Sea and South Africa. Magalhães and Bailey-Brock (2010) redescribed an endemic Hawaiian species, previously been known as *Cirriformiahawaiensis* Hartman, 1956, as a species of *Timarete* species, *T.hawaiensis* (Hartman, 1956). Magalhães et al. (2014) re-examined several multi-tentaculate cirratulid species collected from the Brazilian coast including four *Timarete* species: *T.caribous* (Grube & Ørsted in Grube, 1859), *T.ceciliae* Magalhães, Seixas, Paiva, & Elias, 2014, *T.oculata* (Treadwell, 1932), and *T.punctata* species complex. Seixas et al. (2017) find out that the presence of two cryptic species within *T.punctata* species complex, one species widely distributed from Atlantic and Pacific Oceans and another species from Bahia, South Atlantic Ocean, using the genetic data of COI and 16S sequences.

Within Korean waters, *Timareteantarctica* (Monro, 1930) is the only recorded species of *Timarete* known in this region (Paik 1989). However, Paik (1989)’s description was very brief and lacked a review of the specific characters. While studying the polychaetes from Korean waters, a new species belonging to the genus *Timarete* was discovered. Here, we conducted a detailed examination of this species using methyl green staining pattern (MGSP) and several ontogenetic characters including the segmental origins of tentacular finalents, distributions of the neuroacicular and notoacicular spines, and dorsal shift of branchial finalents as used by previous investigators (Çinar 2007, Magalhães and Bailey-Brock 2010, Magalhães et al. 2014). In this study, the description and illustrations of the new species are provided together with molecular data pertaining to the barcoded regions of the mitochondrial cytochrome c oxidase subunit I (COI) and 16S ribosomal RNA (16S). Additionally, we reviewed and discussed *Timarete* species recorded from East Asia.

### Materials and methods

#### Sampling and morphological observations

Samples were collected from the intertidal rocky bottoms. Specimens were sorted using sieves with a pore size of 0.5 mm, fixed initially with a 5% formaldehyde-seawater solution, and transferred to 85% ethyl alcohol. The characteristics of the whole body were observed with appendages dissected in a petri-dish using a pair of dissection forceps, or surgical knives and needles under a stereomicroscope (SMZ1500; Olympus, Tokyo, Japan). Dissected specimens were mounted onto temporary slides using glycerol or permanent slides using polyvinyl lactophenol solution. Drawings were based on stereomicroscopy and light microscopy (LABOPHOT-2; Nikon, Tokyo, Japan) aided by drawing tubes. Photographs were captured using an image system (LAS V4.7, Leica Microsystems, Heerbrugg, Switzerland). Specimens for scanning electron microscopy (SEM) were dehydrated with a t-BuOH freeze dryer (VFD-21S; Vacuum Device, Ibaraki, Japan). They were mounted on stubs and coated with gold-palladium. SEM observations were conducted using a scanning electron microscope (SU3500; Hitachi, Tokyo, Japan). Type material and additional material examined were deposited at the National Institute of Biological Resources (NIBR) in Incheon, Korea and the National Marine Biodiversity Institute of Korea (MABIK) in Seocheon, Chungcheongnam-do, Korea, respectively.

#### Molecular analysis

Genomic DNA was extracted from the posterior segments of three specimens selected from the additional material using a DNeasy Blood and Tissue Kit (Qiagen, Hilden, Germany) according to the manufacturer’s protocol. The partial sequences of the mitochondrial cytochrome c oxidase subunit I (COI) and 16S ribosomal RNA (16S) from gDNA were amplified using polymerase chain reaction (PCR) with the following primers: LCO 1490 5'-GGTCAACAAATCATAAAGATATTGG-3' and HCO 2198 5'-TAAACTTCAGGGTGACCAAAAAATCA-3' in COI amplification (Folmer et al. 1994) and 16SarL 5'-CGCCTGTTTATCAAAAACAT-3' and 16SANR 5'- GCTTACGCCGGTCT AACTCAG-3' in 16S amplification (Palumbi 1996; Zanol et al. 2010). PCR amplification was conducted in a total volume of 20 µL: 10 µL of 2x DyeMIX-Tenuto (Enzynomics), 0.5 µL of each primer, 1 µL of gDNA, and 8 µL of sterile water. Touchdown-PCR was carried out according to the following cycling program: 94 °C for 5 min, 94 °C for 1 min, 50 °C for 1 min and 72 °C for 1 min, followed by 20 cycles at decreasing annealing temperatures in decrements of 0.5 °C per cycle, followed by 1 min at 94 °C, 15 cycles of 1 min at 40 °C, 1 min at 72 °C, and final extension at 72 °C for 7 min. PCR products were purified with a QIAquick PCR Purification Kit (Qiagen, Chatsworth, CA, USA). Sequences of the new species were obtained using an Applied Biosystems 3730 DNA sequencer. These sequences were aligned with those of other *Timarete* species using Clustal W (Thompson et al. 1994, Larkin et al. 2007) in Geneious Pro v.9.1.8 (Biomatters, Auckland, New Zealand). The genetic distances of the new species from other species and the Maximum likelihood (ML) tree were determined by MEGA v.6.06 (Tamura et al. 2013).

## Systematic accounts

### Family Cirratulidae Ryckholt, 1851

#### Genus *Timarete* Kinberg, 1866

##### Timarete posteria sp. n.

Taxon classificationAnimaliaTerebellidaCirratulidae

http://zoobank.org/E13A2164-7182-4D18-ACD1-80C4F4C1A9B1

Figs 1 , 2 , 3 , 4

###### Material examined.

***Type locality***: South Korea, Gyeongsangbuk-do Province: Pohang-si County, Heunghae-eup, Odo-ri, 36°09’17”N, 129°24’02”E, 13 July 2017, intertidal rocky bottom. ***Holotype***: complete specimen (NIBRIV0000829700). ***Paratypes***: one complete specimen (MABIKNA00146231); one complete specimen (MABIKNA00146236); one complete specimen (MABIKNA00146238); one complete specimen (MABIKNA00146239); one complete specimen (MABIKNA00146245). ***Non-type material***: 16 specimens (13 complete and 3 incomplete specimens), collection details same as type materials; 11 specimens (all complete), South Korea, Gangwon-do Province: Goseong-gun County, Jugwang-myeon, Munamjin-ri, 35°18'41"N, 129°32'33"E, 10 April 2017, intertidal rocky bottom.

###### Diagnosis.

Body with deep ventral groove and distinct segments. Prostomium triangular, without eyespots. Peristomium evenly divided into three annulations. Branchialfilaments one pair per segment, beginning from third peristomial annulus, and gradually shifting to mid-dorsum between chaetigers 30–78; completely shifted branchiae at about one-third distance between notopodium and dorsal midline. Grooved tentacular finalents arising from chaetigers 5–6 and occasionally 6–7 or 7–8. Chaetae including capillaries and acicular spines; notopodial spines 1–4, pale brown in color, from chaetigers 16–45; neuropodial spines 2–4, curved distally, thicker than notoacicular spines, dark brown in color, from chaetiger 24–69.

###### Description.

Holotype: complete, 5.5 cm in length (4.8–13.2 cm in paratypes) and 5.7 mm in maximum width (4.0–6.0 mm in paratypes), with approximately 261 segments.

**Figure 1.**
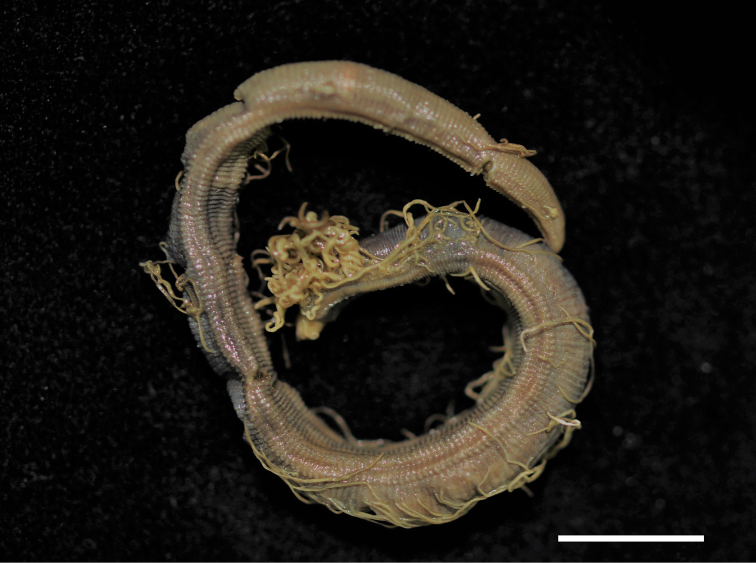
*Timareteposteria* sp. n., paratype (MABIKNA00146236), lateral view. Scale bar: 1.0 mm.

*Body* elongated, rounded dorsally, flattened ventrally, with distinct ventral groove throughout and tapering posterior end. All segments distinct, narrow, crowded throughout body with distinct lateral shoulders. Body color in alcohol pale grey to dark grey, branchiae and tentacular finalents yellowish grey; live specimens with body dark red and branchiae and tentacular finalents light orange. No separate pigmentation on body (Fig. 1).

**Figure 2.**
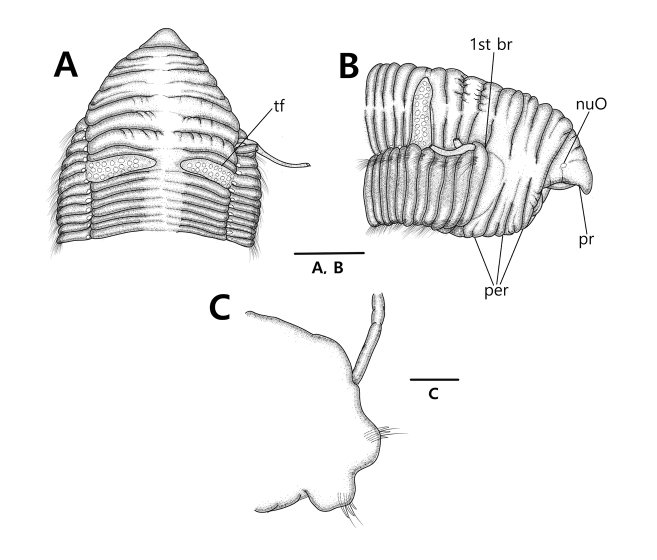
*Timareteposteria* sp. n., **A–B** holotype (NIBRIV0000829700) **C** paratype (MABIKNA00146239) **A** anterior end, dorsal view **B** anterior end, lateral view **C** parapodium of mid-body. Scale bars: 1.0 mm (**A, B**), 0.5 mm (**C**). Abbreviations: branchia (br), chaetiger (ch), nuchal organ (nuO), peristomium (per), prostomium (pr), tentacular finalents (tf).

**Figure 3.**
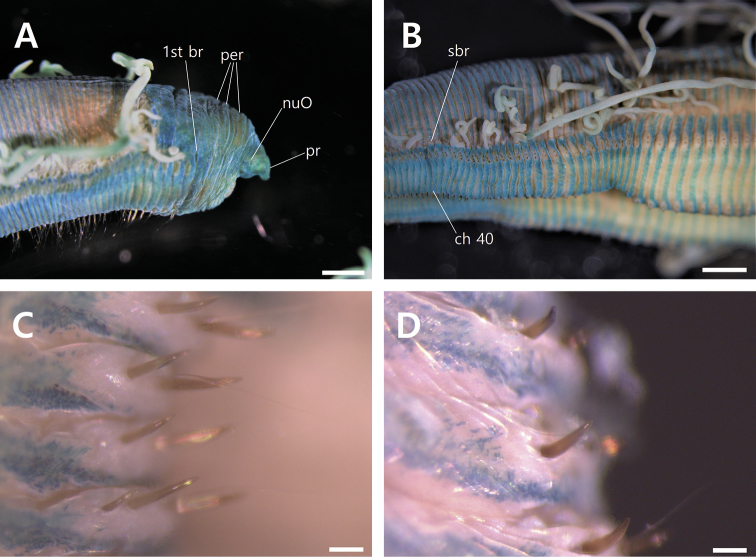
*Timareteposteria* sp. n., **A–D** holotype (NIBRIV0000829700) **A** anterior end, lateral view with MGSP**B** mid-body segments, lateral view with MGSP**C** notopodial spines of mid-body segments **D** neuropodial spines of mid-body segments. Scale bars: 1.0 mm (**A, B**), 0.1 mm (**C, D**). Abbreviations: branchia (br), chaetiger (ch), nuchal organ (nuO), peristomium (per), prostomium (pr), shift of branchial filament (sbr).

*Prostomium* short, triangular, blunt distally, and as long as three anterior chaetigers. Nuchal organs round, present on posterior-lateral prostomial region. Eyespots absent (Figs 2A, B, 3A).

*Peristomium* with three annulations nearly equal in length, longer than prostomium and as long as four anterior chaetigers; second and third annulations with 2–3 lateral wrinkles (Figs 2A, B, 3A).

*Branchialfilaments* one pair per segment, from posterior margin of third peristomial annulus, continuing on most segments except about last ten segments; branchial finalents located just above notopodial ridges in anterior 42 chaetigers (29–77 chaetigers in all specimens examined); then shifting gradually to mid-dorsum forming lateral bulge over notopodia from chaetiger 43 (30–78 in all specimens examined) to near posterior end; fully shifted branchiae located about one-third distance between notopodium and dorsal midline. (Figs 2A–C, 3B).

*Tentacularfilaments* formed two transverse groups separated by median gap and arising on dorsum of chaetigers 5–6 (6–7 or 7–8 in some specimens examined); each group with about 18–21 finalents arranged in 2–3 transverse rows (Figs 2A, B, 3A).

*Parapodia*, notopodia forming lateral shoulders dorsally; noto- and neuropodium widely separated throughout (Figs 2A–C, 3A, B).

**Figure 4.**
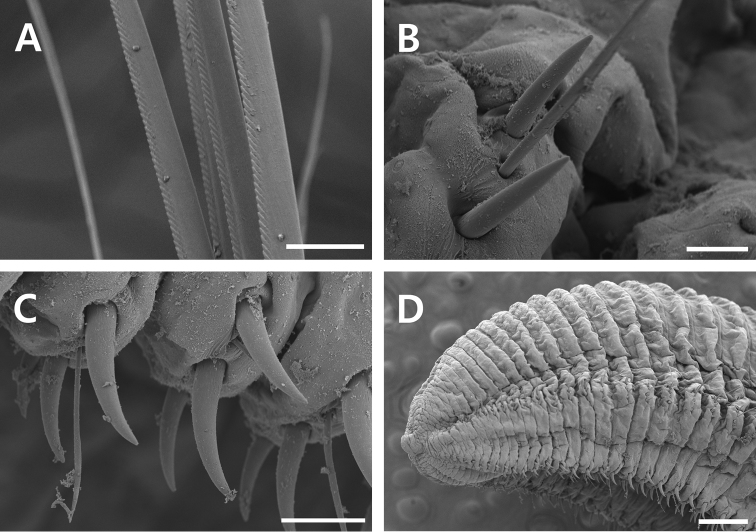
SEM observation of *Timareteposteria* sp. n., **A–D** paratype (MABIKNA00146238) **A** capillary chaetae of anterior chaetiger **B** notopodial spines of posterior chaetiger **C** neuropodial spines of posterior chaetiger **F** pygidium, lateral view. Scale bars: 0.02 mm (**A**), 0.025 mm (**B**), 0.05 mm (**C**), 0.25 mm (**D**).

*Chaetae* including capillaries with serrated edge observed under light microscopy (400x) and SEM observation and acicular spines. Capillary chaetae about 8–10 capillaries arranged in two longitudinal rows in anterior parapodia. Notopodial spines nearly straight, pale brown in color, present from chaetiger 40 (16–45 in all specimens examined); 1–3 spines per segment accompanied by 1–3 companion capillaries from chaetiger 40 to posterior end. Neuropodial spines curved distally, slightly thicker than notopodial spines, dark brown in color, from chaetiger 30 (24–69 in all specimens examined); 2–3 spines per segment with 1–2 companion capillaries from chaetiger 30 to very posterior end (Figs 3C, D, 4A–C).

*Pygidium* with terminal anus (Fig. 4D).

###### Methyl green staining pattern (MGSP).

Body stained with transverse bands on posterior half of each segment forming complete rings. Branchial and tentacular finalents not stained. Prostomium, peristomium, and dorsum of first 3 or 4 chaetigers intensely stained with dark green. Noto- and neuropodial ridges not stained (Fig. 3A, B).

###### Variations.

**Figure 5.**
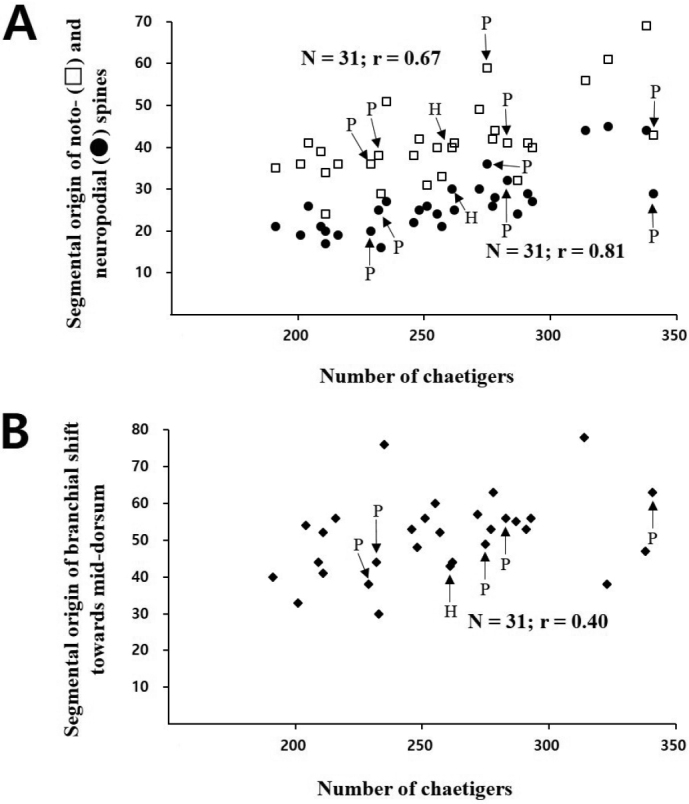
Variation of ontogenetic characters in *Timareteposteria* sp. n. **A** relationship between total number of chaetigers and segmental origin of noto- and neuropodial spines **B** relationship between total number of chaetigers and segmental origin of branchial shift toward mid-dorsum. Abbreviations: holotype (H), paratypes (P).

Several morphological characters in cirratulids are highly variable ontogenetically and a few of them are clearly considered size-dependent in *Timarete* species (Blake 1996, Magalhães and Bailey-Brock 2010, Magalhães et al. 2014). We examined the relationships between the ontogenetic characteristics including the segmental origin of noto- and neuropodial spines and the shift of branchial finalents, and the total number of chaetigers in the new species according to the correlation analyses based on 31 complete specimens (Fig. 5). In *Timareteposteria* sp. n., the segmental origin of neuropodial spines ranged from chaetigers 16 to 45, strongly size-dependent (N = 31, r = 0.81). The segmental origin of notopodial spines varied from chaetigers 24 to 69, with weak size-dependent characteristics if compared with those of neuropodial spines (N = 31, r = 0.67). The dorsal shift of branchial finalents occurred between chaetigers 30 and 78 regardless of the total number of chaetigers in the new species (N = 31, r = 0.40) (Fig. 5). It is known that the appearance of tentacular finalents is generally variable in a few *Timarete* species (Magalhães et al. 2014). In *T.posteria* sp. n., the tentacular filaments always originated in the dorsum of two consecutive chaetigers although their locations were variable: usually on chaetigers 5–6 (in 28 specimens) and occasionally 6–7 (in two specimens) or 7–8 (in four specimens) in all the 34 specimens examined. This variation appears to be somewhat related to body size because the tentacular finalents on chaetigers 7–8 occur in specimens containing more than 300 segments (almost similar to specimens with less than 300 segments on chaetigers 5–6). Further studies with a larger population and more temporal samples are needed to determine a more accurate relationship between the segmental origin of the tentacular finalents and the body size.

###### Etymology.

The epithet of the specific name, *posteria*, is derived from the Latin *posterior*, meaning ‘hind’. This name refers to the shift in the appearance of the branchial finalents from relatively posterior chaetigers. The gender of the genus name, *Timarete*, is feminine and the specific name of this new species is designated as feminine.

###### Habitat and distribution.

This species is a common inhabitant of seagrass beds in the intertidal rocky bottoms and distributed in the East Sea (or the Sea of Japan) of South Korea.

###### Molecular information.

**Figure 6.**
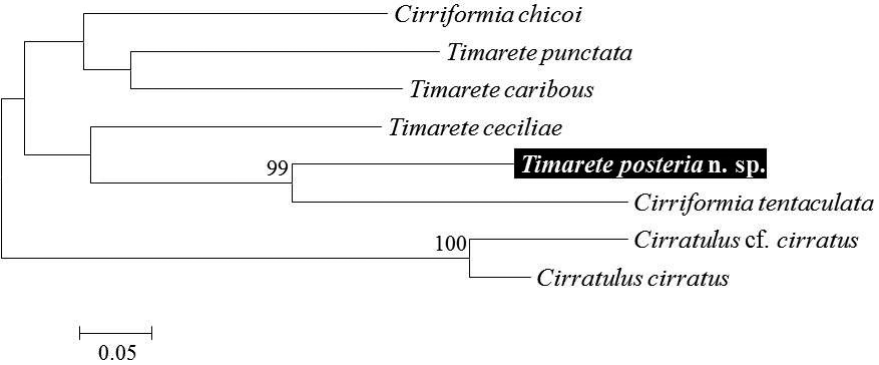
Maximum likelihood (ML) tree for four species of *Timarete* with four other related species in the multi-tentaculate cirratulid genera, *Cirriformia* and *Cirratulus*, inferred from combined dataset with COI and 16S. Numbers above the branch indicate ML bootstrap values from 1000 replication.

In the present study, partial COI sequences each measuring 658 bp in size from five specimens and partial 16S sequence of 519 bp in size from a single specimen were obtained for the genetic analysis of *Timareteposteria* sp. n. They were deposited in GenBank under the accession number MH708229–MH708233 (COI) and MH822840 (16S). The intra-specific genetic distance between five COI sequences was measured according to the Kimura-2-parameter (K2P) model and ranged from 0 to 0.4 %. We carried out the genetic comparison of the new species with three *Timarete* species availabl*e, i*ncluding *T.caribous* (Grube and Ørsted in Grube, 1859), *T.ceciliae* Magalhães, Seixas, Paiva, and Elias, 2014, and *T.punctata* (Grube, 1859) from the Brazilian coast, with COI and 16S sequences previously announced from GenBank (Magalhães et al. 2014). Based on entire genetic data uploaded in GenBank, the inter-specific genetic distances of COI and 16S sequences between the new species and other *Timarete* species were 23.7–26.2 % and 22.2–26.5 %, respectively (K2P distance). We examined the molecular phylogenetic relationship based on the Maximum likelihood (ML) tree using the genetic data available from GenBank on several cirratulids belonging to the multi-tentaculate genera, *Cirriformia*, *Cirratulus*, and *Timarete*, with the new species (Rousset et al. 2007, Hardy et al. 2011, Magalhães et al. 2014, Weidhase et al. 2014, Lobo et al. 2016, Weidhase et al. 2016). The GenBank accession numbers of them are represented on Table 1. In ML tree (Fig. 6), all cirratulid species showed the specific validity by the molecular data of the present study. In generic level, the *Timarete* species including *T.posteria* sp. n. formed a clade with two *Cirriformia* species, *C.chicoi* Magalhães, Seixas, Paiva, & Elias, 2014 and *C.tentaculata* (Montagu, 1808), showing a similar result to the phylogenetic tree of Magalhães et al. (2017). This result suggests that both of the genera *Timarete* and *Cirriformia* are not monophyletic and they are closely related to each other. However, the reality of phylogenetic relationship between *Timarete* and *Cirriformia* still merits further study with more morphological and molecular information of the multi-tentaculate genera.

**Table 1.** GenBank accession numbers for sequences obtained in the present study.

Species	GenBank accession number		Data source
	COI	16S	
*Timareteposteria* sp. n.	MH708229	MH822840	Present study
* Timarete caribous *	KM192177	KM192193	Magalhães et al. 2014
* Timarete ceciliae *	KM192179	KM192195	Magalhães et al. 2014
* Timarete punctata *	KM192188	KM192205	Magalhães et al. 2014
* Cirriformia tentaculata *	KR916808	KT033725	Lobo et al. 2016 (COI)
			Weidhase et al. 2016 (16S)
* Cirriformia chicoi *	KM192165	KM192189	Magalhães et al. 2014
* Cirratulus cirratus *	HM417794	DQ779609	Rousset et al. 2007 (16S)
			Hardy et al. 2011 (COI)
Cirratulus cf. cirratus	KM083601	KT033724	Weidhase et al. 2014 (COI)
			Weidhase et al. 2016 (16S)

###### Remarks.

The major characteristics of *Timareteposteria* sp. n. are mostly similar to those of *Timareteluxuriosa* (Moore, 1904), originally described from Southern California (Blake 1996). Both species share the following morphological features: 1) branchial finalents a single pair per segment, gradually shifting toward mid-dorsum from relatively posterior chaetigers compared to its congeners (the dorsal shift of branchial finalents occurred at about chaetiger 35 when the body with 350 segments in *T.luxuriosa* and occurred at chaetigers 38–78 with more than 300 segments and at chaetigers 30–76 with less than 300 segments in the new species, while that occurred at chaetigers 7–26 in other *Timarete* species); 2) tentacular finalents arising from the dorsum of chaetigers 5–6 (usually 5–6 and sometimes 6–7 or 7–8 in the new species); 3) notopodial spines originating from chaetiger 50 (24–69 in the new species) and pale brown in color; and 4) neuropodial spines originating from chaetiger 31 (16–45 in the new species) and dark brown in color (Blake 1996). However, *T.posteria* sp. n. clearly differs from *T.luxuriosa* in peristomium, notopodial spines, and shifting branchial finalents as follows: 1) the peristomium is evenly divided into three annulations in *T.posteria* sp. n., while that of *T.luxuriosa* comprises one large and three smaller annulations; 2) *T.posteria* sp. n. bears 2–4 neuropodial spines accompanied by a few capillaries in the posterior chaetigers, however, *T.luxuriosa* contains a single neuropodial spine without capillaries in the posterior chaetigers after around chaetiger 90; 3) completely shifted branchial finalents are located at about one-third distance between notopodium and dorsal midline in *T.posteria* sp. n., whereas those of *T.luxuriosa* are positioned at about two-thirds distance (Blake 1996).

The methyl green staining pattern (MGSP), which may be of diagnostic value, is unavailable for many *Timarete* species except for a few species recently described (Imajima and Hartman 1964, Blake 1996, Çinar 2007, Magalhães and Bailey-Brock 2010, Magalhães et al. 2014). Nevertheless, the MGSP of the new species is distinct from the previously described patterns from five *Timarete* species, including *T.caribous* (Grube, 1859), *T.ceciliae* Magalhães, Seixas, Paiva & Elias, 2014, *T.hawaiensis* (Hartman, 1956), *T.oculata* (Treadwell, 1932), and *T.punctata* (Grube, 1859), by a combination of the following features: 1) intense staining of the prostomium, peristomium, and dorsum of the first three chaetigers; 2) the presence of transverse bands forming complete rings in the posterior half of each segment (Magalhães and Bailey-Brock 2010, Magalhães et al. 2014). MGSP is a useful diagnostic feature in *Timarete* species, and additional MGSP information for several *Timarete* species is still required.

## Discussion

*Timareteantarctica* (Monro, 1930) was originally reported from South Georgia in the Antarctic region (Monro 1930, Hartman 1966). It also has been widely reported from Korean and Japanese waters (Imajima and Hartman 1964, Paik 1989). However, this species is clearly distinguished from other *Timarete* species including *Timareteposteria* sp. n. based on having single capillary chaetae and longitudinal rows of tentacular finalents, while other *Timarete* species contain both capillary chaetae and acicular spines, and tentacular finalents arranged in transverse rows (Monro 1930, Imajima and Hartman 1964, Hartman 1966, Paik 1989, Blake 1996, Magalhães et al. 2014). Therefore, generically *T.antarctica* should be referred to the genus *Protocirrineris* Czerniavsky, 1881 based on the chaetal composition and arrangement of tentacular finalents (Blake 1996).

Blake (1996) suggested that the shift of the branchial finalents toward the mid-dorsum in middle and posterior chaetigers is a significant generic diagnostic feature of *Timarete*. However, some *Timarete* species, including *T.japonica* Zachs, 1933, *T.dasylophius* (Marenzeller, 1879), and *T.gibbosa* (Moore, 1903) from East Asia, have been recorded or combined without considering the shift in branchial finalents (Zachs 1933, Imajima and Hartman 1964). Zachs (1933) defined *T.japonica* from the Sea of Japan based on a very brief record without description and illustration. Although the detailed diagnostic features of the species were inadequately dealt with, *T.japonica* is distinctly different to *Timarete* species in the diagnostic features suggested by Blake (1996). Zachs (1933) suggested that the lateral branchial finalents attached almost at the bases of notopodia are a diagnostic feature of *T.japonica*. We think that *T.japonica* may be a species of *Cirriformia* Hartman, 1936, because of the absence of shifting branchial finalents.

Imajima and Hartman (1964) redefined two cirratulid species originally recorded from Japanese waters, which were described as species of the genus *Cirratulus* Lamarck, 1818, as *Timarete* species, *T.dasylophius* and *T.gibbosa*. The single diagnostic feature of *Timarete* species included the tentacular finalents present on the dorsum of two or more chaetigers, which determined the taxonomic status of these two species (Imajima and Hartman 1964). Among these two species, *T.gibbosa* has the shift of branchial finalents, which indicates that a pair of branchiae per segment are located at about midway between the parapodium and the dorsal mid-line except those on anterior segments (Moore 1903). Despite of having shifted branchial finalents, *T.gibbosa* is suspected to be a species of *Cirratulus* based on Imajima and Hartman’s (1964) description indicating that this species has the transverse series of eyespots on the prostomium and the neuropodial spines from chaetiger 1. These characteristic features are commonly observed in *Cirratulus* (Blake 1996, Bottero et al. 2017). Moreover, the shift of branchial finalents, which is one of the representative characteristic features of this species, is also found from *Cirratulus* as well as *Timarete* (Blake 1996, Bottero et al. 2017). The affiliation of *C.dasylophius* to *Timarete* is also questionable because of the lack of reference to the shift of branchial finalents in the original description as well as Imajima and Hartman’s study (Marenzeller 1879, Imajima and Hartman 1964). Furthermore, *T.dasylophius* has the branchial finalents from chaetiger 2 (Imajima and Hartman 1964) while *Timarete* species generally bear those from the last peristomial annulation or chaetiger 1 (Blake 1996, Magalhães et al. 2014). We suppose that Imajima and Hartman (1964) may have overlooked the presence of the scars of branchial finalents present on the peristomium or chaetiger 1.

Consequently, we suggest that three *Timarete* species previously recorded from East Asia, *T.japonica*, *T.dasylophius*, and *T.gibbosa*, are not valid species within the genus yet. Further study with the type materials is needed to verify their generic affiliation. Under a modern view of cirratulid taxonomy, meanwhile, three species among presently known *Timarete* species, *T.anchylochaeta* (Schmarda, 1861), *T.norvegica* (Quatrefages, 1865), and *T.polytricha* (Schmarda, 1861), are still remaining to be designated as valid members of *Timarete* because previous records of them have only poor information with brief descriptions and simple drawings (Schmarda 1861, Quatrefages 1865, Blake 1996, Magalhães et al. 2014). We herein provide a key to the species regarded as valid members of *Timarete*.

### Key to valid species of the genus *Timarete*

**Table:** 

1	Dorsal branchiae abruptly shifted	**2**
–	Dorsal branchiae gradually shifted	**4**
2	Neuropodial spine on posterior segments single	***T.caribous* (Grube & Ørsted in Grube, 1859)**
–	Neuropodial spine on posterior segments more than two	**3**
3	Number of tentacular finalents 7–9	***T.hawaiensis* (Hartman, 1956)**
–	Number of tentacular finalents 15–20	***T.filigera* (Delle Chiaje, 1828)**
4	Branchialfilaments one pair per segment	**5**
–	Branchiae finalents 2–5 pair per segment	***T.perbranchiata* (Chamberlin, 1918)**
5	Branchiae and tentacular finalents with black lateral stripes	***T.punctata* (Grube, 1859)**
–	Branchiae and tentacular finalents without pigmentation patterns	**6**
6	Lateral bulge over notopodia formed in shift of branchiae	**7**
–	Lateral bulge over notopodia not formed in shift of branchiae	**9**
7	Shift of branchiae arising beyond chaetiger 30	**8**
–	Shift of branchiae arising in chaetigers 12–14	***T.nasuta* Ehlers, 1897**
8	Posterior chaetigers with 2–4 neuropodial spines	***T.posteria* sp. n.**
–	Posterior chaetigers with single neuropodial spine	***T.luxuriosa* (Moore, 1904)**
9	Notopodial spines originated from chaetigers 11–23	***T.ceciliae* Magalhães, Seixas, Paiva & Elias, 2014**
–	Notopodial spines originated from chaetigers 57–58	***T.oculata* (Treadwell, 1932)**

## Supplementary Material

XML Treatment for Timarete posteria
